# The fate of drug discovery in academia; dumping in the publication landfill?

**DOI:** 10.18632/oncotarget.28552

**Published:** 2024-01-24

**Authors:** Uzma Saqib, Isaac S. Demaree, Alexander G. Obukhov, Mirza S. Baig, Amiram Ariel, Krishnan Hajela

**Keywords:** drug discovery and development, clinical trials, academia-industry collaboration, translational research, drug database

Drug discovery is a tedious process taking a long time to divulge whether a molecule is efficacious and specific in hitting the target and then to confirm that the potential drug does not cause severe adverse effects [1]. Many drug candidates fail crossing multiple checkpoints of this long journey, they lag in one or several aspects and never move beyond the research bench to contribute to public health. These setbacks make the process of drug discovery very time consuming, expensive, and tedious [2]. This viewpoint is focused on delineating how and why the multi-million research efforts in the field of drug discovery often fail to reach its full potential.

There is no shortage of studies focusing on drug discovery. They are published on a daily basis describing the efforts encompassing conventional and/or modern drug discovery technology, including structure-based drug design (SBDD), virtual screening, high-throughput screening (HTS), Artificial Intelligence (AI), and cell-based screening approaches (Figure 1). However, many drug development strategies are rather fuzzy in their advancement. Thus, there is a big gap between drug “*discovery*” and “*development*”. This part could be attributed to the lack of synergy between Academia and Industry at multiple levels. A significant part of this failure results from the lack of streamlining of drug development process.

**Figure 1:**
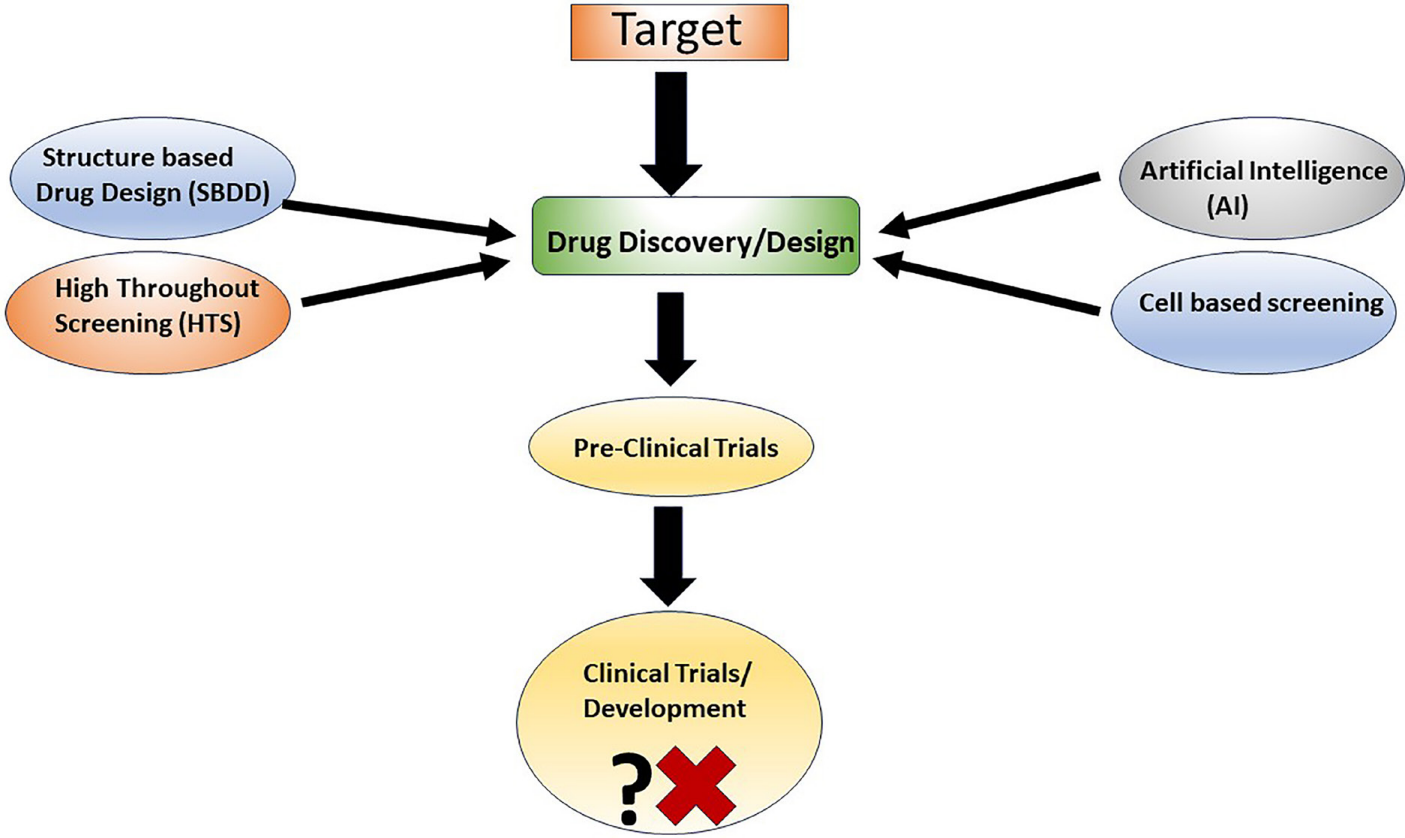
A typical drug discovery and development strategy.

In the current perspective, we discussed why many therapeutic molecules never make it to clinical studies despite being proven efficacious pre-clinically. Additionally, we discussed the possible solutions to overcome this défaut of the drug development process.

Out of more than 2.4 million compounds in the ChEMBL database, about 2.3 million have been tested at the preclinical level (ChEMBL – browse compounds – max phase - preclinical). Most of these compounds have been in the preclinical stage for more than several years or even decades. This essentially means that they are not intended for further clinical testing and hence never progressed beyond this. Only a handful of compounds crossed this stage and entered the clinical study phase [3]. This fact clearly indicates that there is a huge gap between drug discovery and development. We obtain similar or even more compelling results from a search in PubMed. A search for drug discovery efforts during the past few decades indicates that more than 90% research articles describing drug discovery or design stop at the stage of preclinical studies and never move forward to clinical studies. Thus, these potential compounds have a grave fate in terms of drug development suitable for clinical trials. For example, currently, there are >15,000 entries in PubMed for cancer drug discovery over the past 2 decades. This number multiplies exponentially if we take research beyond this time frame or extend the disease list. This data points where the problem lies. It is no doubt that multiple teams across the world are involved in drug discovery research in every area of human disease and quality of life. While many of them reach a significant stage of success, the majority fail to prove the drugs’ efficacy due to the lack of means of providing clinical assessments [4]. Another example is the patent database, which is also overloaded with compounds ‘indicated’ for disease intervention, but most of these compounds never enter any clinical trials in a practical timeframe. Thus, often, it is not that the drugs are failing in clinical trials, but rather they are not exciting the interest of clinical investigators at large drug companies and do not pass traditional benchmarks that make them “a profitable investment.”

This leaves us with several unanswered questions, such as whether the majority of potentially promising compounds which never make it to clinical trials are ill-fated because the investigators never make an effort to progress them for further development or whether the investigators do not find the ‘right’ funding route to proceed further?

There are several factors that contribute to this problem. One reason is that the academic settings almost all over the world, require researchers to publish as many articles as possible in a peer-reviewed journal rather than encouraging them to make efforts to complete the drug discovery process by beginning a clinical study. Of course, some drugs may be unfit for clinical work due to their adverse effects or lack of specificity, but still, there are many promising candidates that get stuck on research benches in the wet labs or academic economic enterprises. Secondly, many investigators lack the experience of academia-industry collaborations and the “know-how” on what makes a compound industry-friendly which could help them navigate smoothly in the transition from discovery to development.

Research in academia is mostly publication-oriented and there are very few investigators who come in-synchrony with Industry to push their developed compounds further. Now, this drawback could mostly be attributed to two main reasons:

(1) In the academic environment, the researchers find it easy to publish and move on to another study rather than waiting for any pharmaceutical companies to test their molecule clinically. They find it cumbersome and complicated to approach and collaborate with Industry for clinical development of their potential lead compounds.

(2) Another obvious downfall in this situation is the lack of funding from government organizations that support academic research for clinical studies. While there are many investigators who are awarded millions in grants for research, most of the funds are spent on consumables and manpower, leaving them with insufficient funds to continue their studies for clinical development. Moreover, some funding agencies foster basic science over translational research and would rank translational efforts as less favorable to be funded. Although there are a handful of developed countries like the USA and UK which allocate funding specifically for clinical studies and trials, the rest of the world is still at the stage where drug candidates remain buried in the academic literature and never progress to clinical testing.

Recent claims suggest that artificial intelligence (AI)-driven drug discovery may accelerate new drug design and implementation [5–7]. However, it is yet to be seen whether all drugs discovered through AI technologies would be progressing towards clinical trials. Because no matter how a drug is discovered, it must eventually pass the pre-clinical and clinical hurdles to be a proven fit for disease intervention. Will the drugs retrieved through AI be again just restricted for publication purposes just like their predecessors discovered through conventional methods? This remains to be seen as AI for drug discovery is just in its budding phase, and we have to wait further to see its complete long-term impact.

Unfortunately, a large portion of the global drug discovery output is not translated in clinical drug development. It is unfortunate that a plethora of manuscripts describing preclinical studies of drug early development would remain untapped potentially for years to come, as very little measures have been taken to address this issue, so far! Only in very sparse cases, pharmaceutical companies cherry-pick a compound from an academic drug development output for further testing, leading to a risk averse strategy in industry with disproportional development of drugs that are already at later clinical stages [8]. On the other hand, the government agencies from many developed and some developing countries are emphasizing and boosting Academia-Industry programs which specifically foster clinical testing and drug development efforts. Additionally, there are a few international funding agencies whose focus is to promote translational research. It is noteworthy that the odds of bringing a drug to the clinic is enhanced significantly when its mechanism of action is known, at least in part. Therefore, basic research and clinical development go hand-in-hand in successful drug development [9, 10].

## What is next?

Evidently, a more robust strategy should be developed to use the full potential of academia drug discovery efforts so that investigators could move the already available drugs to the clinic. This would help to justify the time, effort, and money which have been already spent on the discovery and preclinical testing of drugs in the early pipeline. An international database which contains information on whether a drug has been explored clinically will be helpful and should also provide information on earlier stages of development. For example, if a drug has been identified *in silico* and only a few cell-based assays have been done to prove its efficacy, then this should also be included in the database. Similarly, information on whether a drug has been identified through HTS or AI should also be available. This would enable investigators in next-generation pharmacology to pull specific compounds from the database and perform robust preclinical and clinical testing, with the new information fortifying the knowledgebase. If there is a glaring reason to not pursue the molecule further, then these drugs should be clearly indicated as “discontinued due to adverse effect on…”. Alternatively, the molecule may be listed as “*available* *for further clinical research*” for clinical investigators globally. This would lead to easy identification of molecules to be picked by researchers for further preclinical and clinical testing and would curb unnecessary duplicative studies for compounds which are similar to the ones already identified or are just as efficacious as the ones which are available but not clinically tested. This would save lots of resources for the investigators working on similar areas of research. The investigators would rather pick a drug(s) that has already passed critical preclinical tests and could utilize his/her funds to streamline its development in clinical settings. Remarkably, the National Institutes of Health, the major funding agency supporting extramural translational and clinical research in the USA, has recently pioneered the implementation of the Data Management and Sharing (DMS) policy (https://www.nih.gov/about-nih/who-we-are/nih-director/statements/nih-implements-data-management-sharing-policy) that is aimed at maximizing public sharing of the raw scientific data. This critical policy emphasizes that public data sharing is the foundation for accelerating scientific discoveries in all areas of research.

We believe that funding agencies around the world should consider adopting similar policies and should work on establishing an internationally governed database to foster drug discovery.

In conclusion, we would like to emphasize the importance of utilizing and streamlining our drug discovery efforts towards the clinical usefulness-oriented direction. The rate of failure of drug development programs makes it important to utilize public funds in a more sustainable and efficient way to gain maximum benefit. The documentation of each and every ‘potential’ drug investigated anywhere in the world in a database would be the best starting point to rectify this problem. Such a database should include all how’s, when’s, where’s, what’s, and why’s of the specific drug discovery investigation. Most importantly, it should include what preclinical essays and experimentation have been done previously and what remains to be done to move the specific molecule to clinical trials. This would allow for investigators with new interest in a specific compound to start from where their predecessors have left.

Another important element is that the government organizations should provide more funding specific to clinical research and to investigators with promising results with respect to drug discovery. This would solve two major problems, namely: (1) the funds can be specifically utilized for identified successful drug development programs, and (2) the new discovered drugs, after passing all necessary clinical trials, can then be made available to common people at a more reasonable price.

Lastly, fruitful efforts to bring more drugs from bench to bedside could only be possible if we do not leave them ‘midway’!
